# Pulmonary sarcoidosis complicated with pulmonary cryptococcosis: a case report

**DOI:** 10.3389/fmed.2026.1822801

**Published:** 2026-05-05

**Authors:** Shixuan Wang, Xi Wang, Kunyan Sun, Zhe Jin, Jing Ma

**Affiliations:** Department of Respiratory and Critical Care Medicine, Peking University First Hospital, Beijing, China

**Keywords:** *Cryptococcus*, fungal infection, lung biopsy, pulmonary cryptococcosis, pulmonary sarcoidosis

## Abstract

Pulmonary sarcoidosis is an idiopathic granulomatous disorder primarily affecting the lungs and mediastinal lymph nodes. Pulmonary cryptococcosis, an opportunistic mycosis caused by *Cryptococcus* species, may occur concurrently with sarcoidosis, presenting substantial diagnostic challenges, particularly in treatment-naïve patients. A 63-years-old previously healthy female presented with insidious-onset dyspnea and low-grade fever. Chest computed tomography (CT) showed mediastinal and hilar lymphadenopathy, accompanied by small nodules in the right lower lobe. She was diagnosed with pulmonary sarcoidosis at a local hospital and started on prednisone, with symptomatic improvement. However, follow-up imaging showed enlargement and cavitation of the right lower lobe nodules. Admission laboratory tests, including inflammatory markers and fungal serology, were all unremarkable. Metagenomic next-generation sequencing (mNGS) of bronchoalveolar lavage fluid (BALF) identified sequences of *Cryptococcus neoformans*. Histopathological examination of mediastinal lymph node specimens confirmed the presence of non-necrotizing granulomas, which is consistent with a diagnosis of sarcoidosis. Meanwhile, the right lower lobe lung biopsy revealed positive staining for *Cryptococcus*. The patient was treated with fluconazole, resulting in radiological resolution. This case highlights the importance of considering pulmonary cryptococcosis as a potential complication in treatment-naïve sarcoidosis patients who present with abnormal chest shadows. Underlying immune dysregulation in sarcoidosis may obscure both clinical and radiological findings, thereby complicating the diagnostic process.

## Introduction

Sarcoidosis is a multisystem granulomatous disease characterized by the formation of non-caseating granulomas in affected organs, mainly involving the lungs and thoracic lymph nodes (1). The immune mechanism of this disease is centered on a T-helper response, in which CD4 lymphocytes and activated macrophages accumulate in affected organs, thereby inducing the formation of granulomas (2). Despite much research over the past decade, the cause of sarcoidosis remains elusive.

Pulmonary cryptococcosis, an opportunistic fungal infection caused by *Cryptococcus* species, most commonly occurs in immunosuppressed individuals, but may also develop in immunocompetent patients following exposure to high-risk environments (3). *Cryptococcus* is an encapsulated yeast with a worldwide distribution and is ubiquitous in the environment, including pigeon guano, soil, and plant debris (4). Humans are usually infected by inhaling dust containing fungal spores.

The coexistence of cryptococcal infection in untreated sarcoidosis is an uncommon phenomenon that poses considerable challenges to clinical diagnosis. In particular, cryptococcal infection is prone to being overlooked when it presents as small nodules in the early stage. Herein, we report a case of pulmonary sarcoidosis complicated with pulmonary cryptococcosis at the initiation of disease onset, and the coexistent pulmonary cryptococcal infection progressed during glucocorticoid treatment.

## Case presentation

**FIGURE 1:**
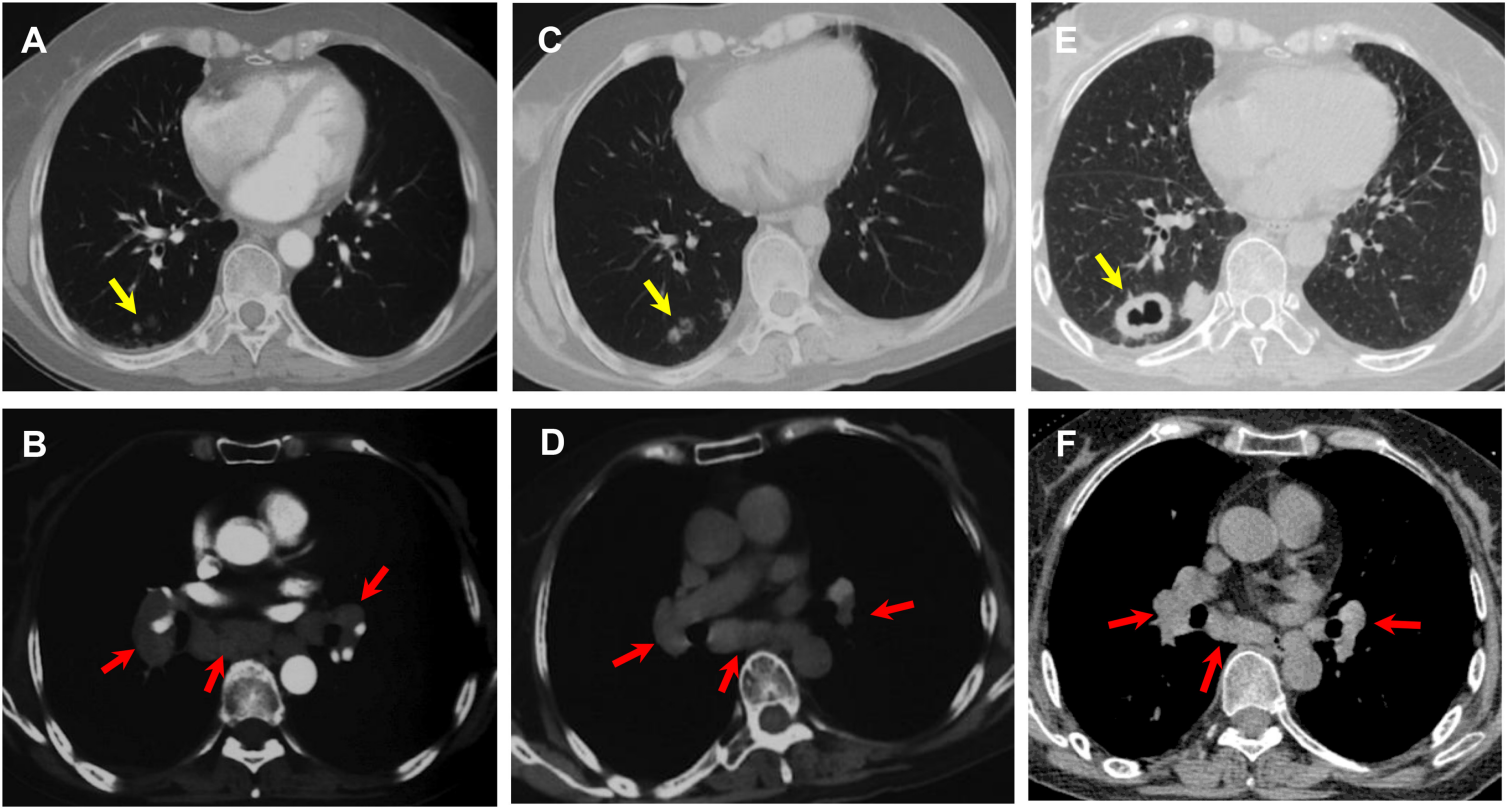
**(A,B)** Chest CT scan at the onset of disease showing mediastinal and hilar lymphadenopathy (red arrows), along with small nodules in the right lower lobe (yellow arrows). **(C,D)** Chest CT scan during glucocorticoid treatment demonstrating the lymph nodes decreased in size (red arrows), while nodules in the right lower lobe increased in size (yellow arrows). **(E,F)** Pre-admission outpatient chest CT scan revealing the nodular shadows progressed in size with cavity formation (yellow arrows).

A 63-years-old previously healthy female was admitted to the Department of Respiration due to abnormal lung lesions. Four months prior, she developed insidious onset of shortness of breath and fever, with body temperature fluctuating between 37°C and 38°C. Chest computed tomography (CT) performed at the initial onset revealed mediastinal and hilar lymphadenopathy (Figure 1B), accompanied by multiple small nodular opacities (4–6 mm in diameter) in the right lower lobe (Figure 1A). Upon initial evaluation at a local hospital, the patient underwent bronchoscopy, with bronchial brushing cytology and microbiological testing for acid-fast bacilli, fungi, and malignant cells all returning negative. She was diagnosed with pulmonary sarcoidosis, and corticosteroid therapy with prednisone was initiated at a daily dose of 40 mg. Her symptoms gradually resolved, and the glucocorticoid was tapered and discontinued after 3 months. Chest CT during glucocorticoid treatment (2 months prior to the current admission) showed a reduction in lymph node size (Figure 1D), while the nodules in the right lower lobe increased to 8–10 mm in diameter (Figure 1C). A pre-admission outpatient chest CT demonstrated a further increase in the size and number of nodules, with the development of cavitation (Figure 1E), as well as persistent mediastinal lymphadenopathy (Figure 1F). Past medical history revealed no underlying diseases. The patient is a retired teacher residing in an urban area, with no history of exposure to pigeons, other avian species, or any other animals.

On admission, the patient was in good general condition without complaints of discomfort. Physical examination revealed no abnormal findings. Complete blood counts, white blood cell differentials, C-reactive protein and serum pro-calcitonin were all within normal ranges. Serum tests, including (1-3)-β-D-glucan assay, galactomannan antigen, cryptococcal antigen, and tuberculous interferon-γ release assay, were all negative.

**FIGURE 2:**
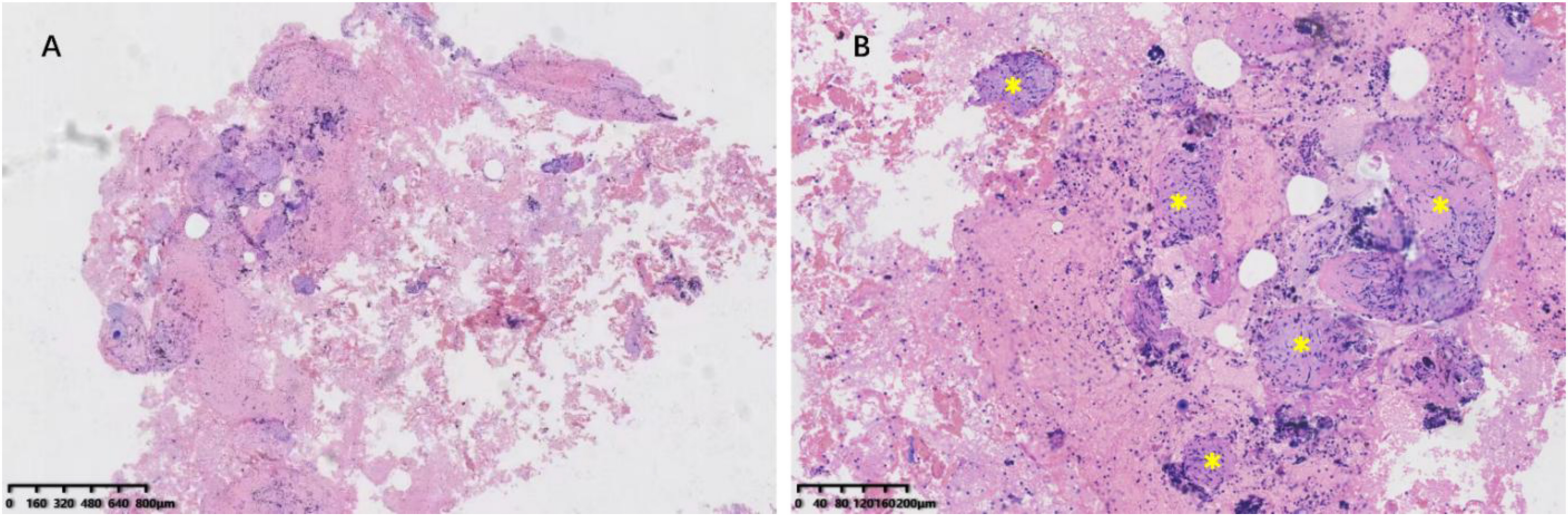
Pathological findings from the mediastinal lymph node. **(A)** Hematoxylin and Eosin (H&E) staining at 40× showing an overall view of the tissue obtained. **(B)** H&E staining at 200× demonstrating non-necrotizing granulomas composed of epithelial cells and multinucleated giant cells (yellow asterisks).

**FIGURE 3:**
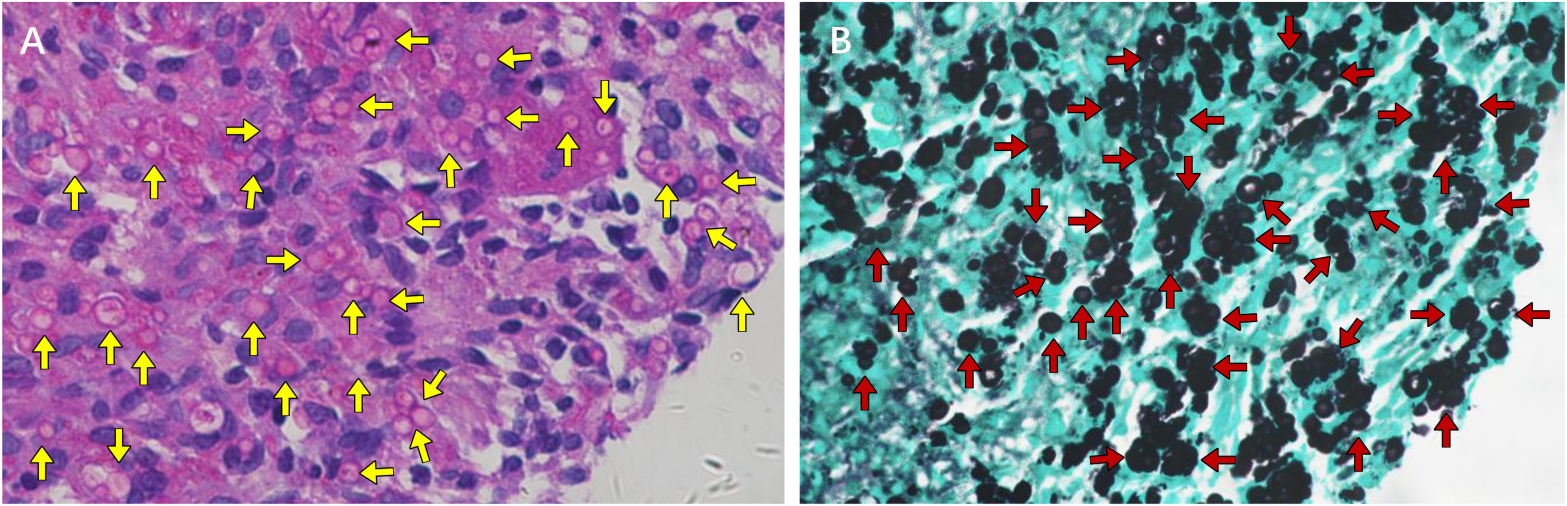
Special stains of the tissue obtained by lung biopsy. **(A)** PAS staining showing *Cryptococcus* organisms appearing as circular vacuoles of varying sizes with purple-red cell wall (yellow arrows). **(B)** GMS staining demonstrating numerous *Cryptococcus* organisms with black-stained cell walls (red arrows).

**FIGURE 4:**
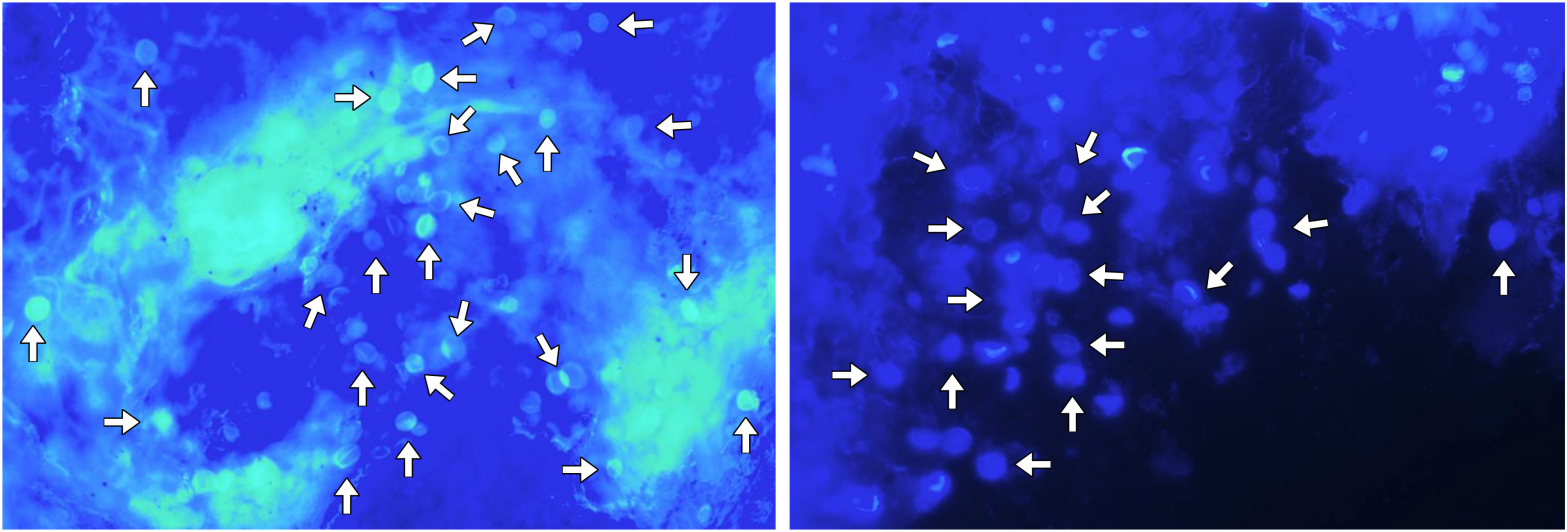
Fungal fluorescent staining of the lung biopsy tissue showing abundant circular yeast-like cell bodies of *Cryptococcus* (white arrows).

The patient underwent bronchoscopy under general anesthesia after admission. Metagenomic next-generation sequencing (mNGS) of bronchoalveolar lavage fluid (BALF) from the superior segment of right lower lobe detected 1,602 sequences of *Cryptococcus neoformans*. Transbronchial needle aspiration biopsy of the mediastinal lymph node showed formation of non-necrotizing granulomas composed of epithelioid cells and multinucleated giant cells, findings highly consistent with sarcoidosis (Figure 2). Pathological examination of lung biopsy specimens from the right lower lobe lesions showed numerous scattered *Cryptococcus* organisms amid inflammatory cell infiltration. Periodic acid-Schiff (PAS) staining demonstrated *Cryptococcus* organisms appearing as circular vacuoles of varying sizes with purple-red cell walls (Figure 3A). In contrast, Grocott-Gomori methenamine-silver (GMS) staining demonstrated numerous yeast-like cell bodies with clearly delineated black-stained cell walls against a light green background (Figure 3B). Additionally, round spores of varying sizes were also identified by fluorescent staining (Figure 4).

**FIGURE 5:**
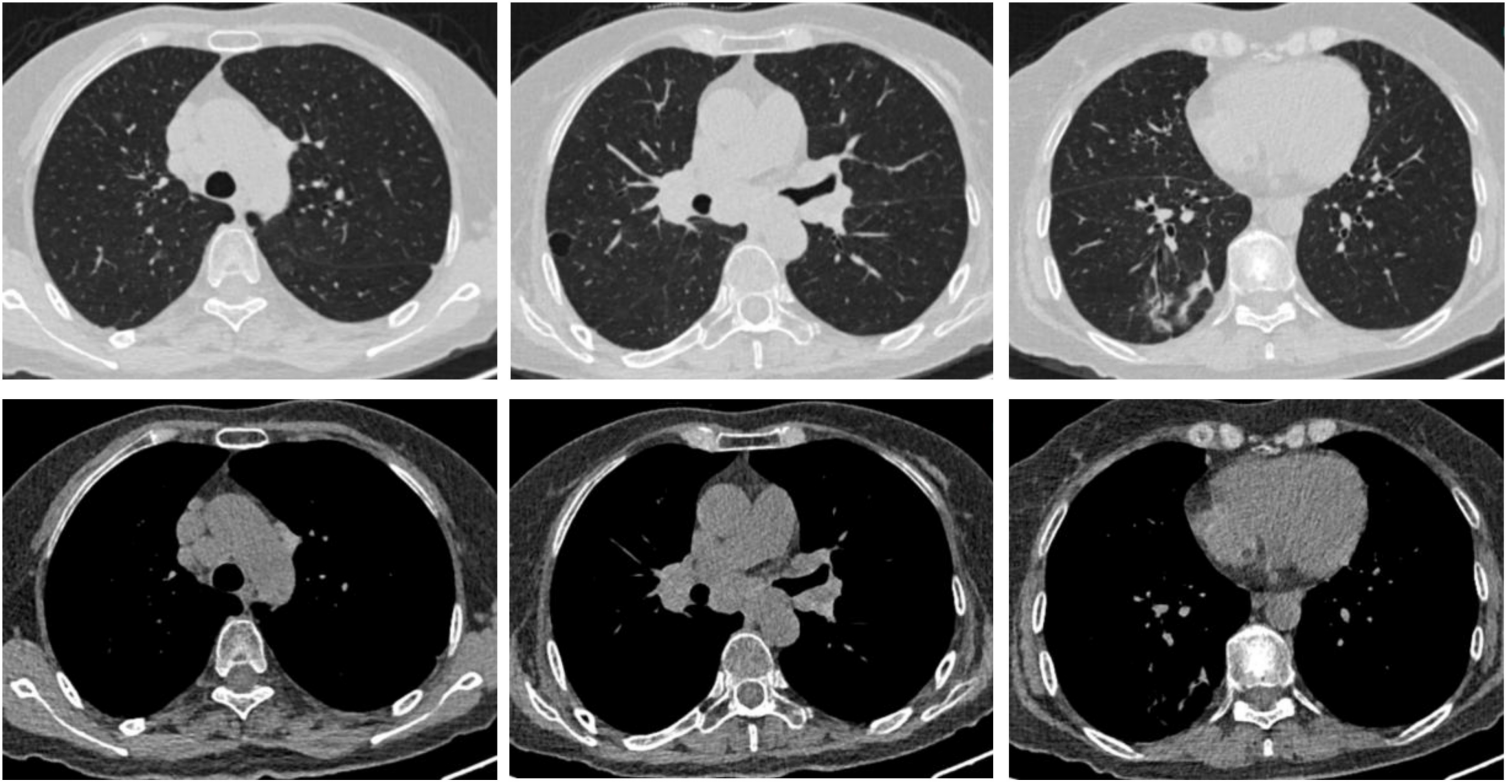
Chest CT scan performed at 6 months after initiation of antifungal treatment demonstrating marked absorption of the cavitary lesions.

Cryptococcal screening for the central nervous system was negative. The patient was ultimately diagnosed with pulmonary cryptococcosis, along with an underlying condition of pulmonary sarcoidosis. She was administered fluconazole for antimicrobial therapy, and a follow-up chest CT performed at 6 months after initiation of antifungal treatment demonstrated marked resolution of the pulmonary lesions (Figure 5).

## Discussion

Our case enriches the literature by documenting a rare comorbidity of pulmonary sarcoidosis complicated with pulmonary cryptococcosis, a clinical entity that poses considerable diagnostic challenges, particularly in treatment-naïve sarcoidosis patients. In this case, we present novel clinicopathological evidence supporting the coexistence of these two conditions.

Pulmonary cryptococcosis is caused by inhalation of dust or particulate matter contaminated with *Cryptococcus*, which is frequently associated with excreta from birds, especially pigeons (3). However, no history of exposure to pigeons or their excreta was identified in the present case. The radiological findings of pulmonary cryptococcosis can be diverse and may mimic many other diseases such as bacteria and mycobacteria infection, malignancy, organizing pneumonia, infarction, etc., (5). Nodules distributed in the peripheral area are the most common appearance of pulmonary cryptococcosis on chest CT. The nodules are more often solitary and with well-defined appearances. The halo sign and fine spicules can be observed, but not very commonly, about 10%–40% by prior studies (6). The diagnosis of pulmonary cryptococcosis primarily depends on histopathological examination, culture-based methods and specific cryptococcal antigen detection. *Cryptococcus* typically appears as narrow-based budding yeasts (4–10 μm) surrounded by thick capsules under the microscope. PAS and GMS staining can better display the cell bodies of *Cryptococcus* and improve the detection rate (6). Fungal fluorescent staining allows for more convenient and intuitive microscopic visualization of fungal pathogens (7). Novel molecular biology techniques, such as mNGS, have been maturely applied in the testing of clinical samples including BALF and cerebrospinal fluid (8, 9).

We identified two case reports in the prior literature documenting concomitant cryptococcal infection in treatment-naïve patients with sarcoidosis in previous literature. Specifically, Shin-ichi Nureki reported a case of a 63-years-old woman who diagnosed with sarcoidosis through scalene lymph node biopsy and the patient didn’t receive any therapy for sarcoidosis (10). During follow-up, chest CT showed progression of nodules in the left lower lobe, which was ultimately confirmed as cryptococcal infection by bronchoscopic biopsy. Another case reported by Bo Zhou also identified concurrent pulmonary cryptococcal infection and sarcoidosis at initial presentation, in the absence of steroid use or immune suppression (11). Compared with these previous reports, our case further enriches the available clinical evidence by providing detailed sequential imaging data that clearly illustrate the presence of cryptococcal infection prior to corticosteroid administration. Moreover, our case presents high-quality, well-documented pathological sections and fungal fluorescence staining images, which offer straightforward and reliable evidence for the definite diagnosis of sarcoidosis and cryptococcosis.

A literature review carried by Girard et al. documented 85 case reports of opportunistic infections complicating sarcoidosis between 1966 and 2004 (12). The study indicated that cryptococcosis was the most frequently reported opportunistic infection in sarcoidosis, accounting for 48.2% (41 cases), followed by nocardiosis (8 cases, 9.4%), non-tuberculous mycobacterial infection (7 cases, 8.2%), pneumocystis (6 cases, 7.0%), histoplasmosis (6 cases, 7.0%), and aspergillosis (4 cases, 4.7%). Another retrospective study conducted by Bernard et al. reported 18 cases of cryptococcosis complicating sarcoidosis and found that one-third of the cases occurred in treatment-naive sarcoidosis patients (13). These aforementioned case reports and studies suggest that sarcoidosis may be prone to cryptococcal infection, which is largely unrelated to immunosuppressive therapy.

The exact causal relationship between sarcoidosis and cryptococcosis has not yet been definitively established. Some studies hypothesize that fungal infections, such as *Coccidioides* and *Cryptococcus*, may act as precipitating factors for sarcoidosis (14). However, more studies suggest that the immune abnormalities existing in sarcoidosis predispose individuals to cryptococcal infection (15). T lymphocyte-mediated immune responses play a central role in the defense against cryptococcal infection (16). Despite extensive local inflammation activation, sarcoidosis presents an “immune paradox,” as indicated by CD4 T lymphocyte dysfunction and impaired immune responses to foreign antigens. Immunological studies performed in patients with sarcoidosis have demonstrated that these individuals exhibit depressed cell-mediated immune responses to cryptococcal antigens *in vitro* (17). Besides, some researchers have noted that the development of opportunistic infections in untreated sarcoidosis should prompt consideration of underlying primary immunodeficiency, which may contribute to the onset of both granulomas and infections (18). Further research is therefore warranted to elucidate the underlying relationship between sarcoidosis and cryptococcal infection.

The treatment options and antifungal course for pulmonary cryptococcosis depend on the host’s immune status, the affected organs and the severity of the disease, whether localized in the lung, or spread to the central nervous system, or generally disseminated (3). Fluconazole is the first-line treatment for asymptomatic, mild, and moderate pulmonary cryptococcosis. For patients presenting with severe symptoms, diffuse infiltrates, or concomitant central nervous system involvement, induction therapy with a combination of an amphotericin B formulation and 5-flucytosine should be administered (4).

## Conclusion

In this study, we report a case of pulmonary sarcoidosis complicated with pulmonary cryptococcosis at the initiation of disease onset, and the coexistent pulmonary cryptococcal infection progressed during the glucocorticoid treatment. As a single case report, the findings mainly serve as a valuable clinical reminder that clinicians should maintain a high suspicion for opportunistic infections such as cryptococcosis in untreated sarcoidosis patients who present with atypical radiologic progression. Our case also underscores the critical role of lung biopsy and mNGS testing in achieving a precise diagnosis, which is essential for the prompt initiation of appropriate therapy.

## Data Availability

The original contributions presented in this study are included in the article/supplementary material, further inquiries can be directed to the corresponding author.
